# Auxological and endocrinological features in internationally adopted children

**DOI:** 10.1186/s13052-020-00832-5

**Published:** 2020-06-10

**Authors:** Stefano Stagi, Valeria Papacciuoli, Djibril Boiro, Chiara Maggioli, Niane Ndeye Ndambao, Stefania Losi, Elena Chiappini, Sonia Toni, Ousmane Ndiaye

**Affiliations:** 1 Health Sciences Department, University of Florence, Anna Meyer Children’s University Hospital, Viale Pieraccini 24, 50139 Florence, Italy; 2 Service Universitaire de Pediatrie, UCAD, Dakar, Senegal

**Keywords:** Adopted children, Growth, Growth chart, Puberty, Precocious puberty, Bone age, Congenital hypothyroidism, Iodine

## Abstract

In internationally adopted children disorders of linear growth, puberty development, thyroid function, and bone metabolism are frequently reported. It is important that these children receive careful auxological and endocrinological evaluations and follow-up.

Pediatricians and other healthcare providers should be aware that auxological and endocrinological problems are common in newly arrived international adoptees.

## Key notes

- Children who are adopted internationally should be assessed for linear growth, puberty development, thyroid function, and bone metabolism disorders.
- A close auxological and endocrinological evaluation with follow-up is mandatory in these children.
- Pediatricians and other healthcare providers should be aware that auxological and endocrinological problems are common in newly arrived international adoptees.

## Introduction

Approximately 260,000 international adoptions take place worldwide each year [1]. The countries which welcome the highest numbers of children are the USA, France, Spain and Italy where nearly 2000 international adoptees arrive every year [2].

In recent years there have been important changes regarding the volume of adoptions, the countries involved and the characteristics of families deemed suitable to adopt [1]. The living conditions of children in their countries of origin are very variable. Most live in orphanages, where they may experience emotional abandonment, physical neglect and malnutrition and be exposed to infectious diseases (ex. tuberculosis and parasitic infections) [3, 4]. The history of these children may be positive for pre- and perinatal complications, such as exposure to drugs and alcohol during gestation, absence of perinatal care, low birth weight, and prematurity [3, 4]. Often a combination of these factors leads to auxological or endocrinological problems and it is important to bear this in mind when the children arrive in their country of adoption [5]. Medical problems are reported in more than 40% cases [6]. Early pubertal development, chronic malnutrition and low weight are frequently reported conditions [7, 8]. Initial assessments are often complicated by the absence of up to date growth charts for specific countries and the lack of neonatal screening programs or paediatric care in the countries of origin.

In this review we evaluate the main auxological and endocrinological problems encountered in international adoptees in order to shed light on those that are most frequent. We hope to provide some useful suggestions for looking after these children.

### Growth and growth disorders

The assessment of growth is the most useful tool for defining the health of a child. By monitoring growth, we can evaluate a child’s nutritional status. The Tanner scale allows us to determine whether pubertal development is at an appropriate stage and diagnose any growth disorders with a non-nutritional basis [9].

Internationally adopted children may present growth suppression as a consequence of institutional care in their countries of origin [10]. Factors which negatively impact growth include neglect and chronic malnutrition due to a lack of sufficient personnel to meet the needs of the children [11]. Moreover, parasite infections can result in iron deficiency and a reduction in the absorption of micro- and macronutrients [10, 12, 13].

Neglect during early childhood seems to slow growth rates in height and weight for children, suggesting that early childhood is a critical period for a child’s growth in later life [14]. In fact, institutionalized children often experience emotional and nutritional deprivation that precludes adequate environmental stimulation and nutrition, essential elements for normal growth [15]; in these subjects, growth delay has been termed psychosocial dwarfism. Moreover, stress from psychological harassment has been shown to lead to abnormal circadian rhythms and suppression of growth hormone release [16].

Post-placement recovery has been widely documented. In fact, most adoptees demonstrate excellent catch-up growth within six months of adoption. If growth failure persists after 6 months of appropriate caloric intake, nutrition-independent causes should be considered [17]. However, little is known about how long children take to recover normal growth patterns, especially if we consider that pre-adoptive experiences are diverse [18]. Generally, following adoption, children undergo a variable catch-up growth, in part due to normalization of the growth hormone system [19].

However, some data suggest that the length of this catch-up period may depend on the children’s countries of origin and that there may be significant auxological variations. For example, children adopted from Ethiopia or Eritrea often reside for relatively long periods of time with relatives prior to institutionalization and exhibit few behavioural problems on arrival. In general, they have good growth, and less severe developmental delays [20]. It can be difficult to accurately establish the child’s age which may to lead to mistakes, for example a misdiagnosis of precocious puberty and inappropriate pharmacological treatments. Unfortunately, there are no accurate tests for determining age. Malnutrition and deprivation may affect assessments of bone age, whereas the onset of puberty and improved nutrition may increase bone density.

Children adopted from Romania and other Eastern European countries have often been placed in institutions with very low quality of care [11] and children adopted from Poland and the former Soviet Union have a high risk of presenting a fetal alcohol spectrum disorder (FASD) which may go undiagnosed [21]. Adoptive parents and professionals need to be aware of the potential consequences of prenatal exposure to alcohol [21, 22] [Table 1]. Guatemalan and Chinese adoptees display similar overall patterns of growth and developmental delays as seen in other groups of internationally adopted children, although not as severe [23, 24].

**Table 1:** Criteria for the Diagnosis of Fetal alcohol spectrum disorders (FASD)

Terminology	Diagnostic Features
Fetal alcohol syndrome (FAS)	All of the following criteria: - three facial abnormalities (ie, smooth philtrum, thin vermillion border, and small palpebral fissures). Midface hypoplasia, micrognathia, microcephaly or epicanthal folds may also be observed; - growth deficiency (height and/or weight ≤ 10th percentile at any age) - structural, neurologic, or functional central nervous system (CNS) abnormalities - prenatal exposure to alcohol (PAE)^a^
Partial FAS	- some (but not all of the physical features of FAS) (see above) - CNS damage (structural, neurologic, and/or functional impairment) - confirmed prenatal exposure to alcohol
Alcohol-related birth defects	Individuals who do not have the facial characteristics of full FAS - Significant birth defects affecting the heart, eyes, kidneys, and/or bones resulting from PAE - Hearing may also be affected - Usually do not meet criteria for CNS structural or functional abnormalities - Confirmed prenatal exposure to alcohol
Alcohol-related neurodevelopmental disorder	Cluster of symptoms that may include intellectual disabilities as well as challenges with behaviour and learning resulting from PAE May also have a CNS anomaly Often perform poorly in school and have difficulties with maths, memory, attention, judgment, and impulse control Confirmed prenatal exposure to alcohol
Neurobehavioral disorder associated with prenatal alcohol exposure (ND-PAE)	Impairment of neurocognition, self-regulation, and adaptive functioning - Combines deficits in these 3 areas in conjunction with evidence of PAE, childhood onset of symptoms, and significant distress or impairment in social, academic, occupational, or other important areas - Confirmed prenatal exposure to alcohol

CNS, central nervous system. ^a^Not necessary if the first 3 features are present. Adapted by [25].

As well as presenting stunted growth, malnourished children often present retarded bone maturity comparable to children affected by diseases such as coeliac disease, inflammatory bowel disease and hormonal deficiency. If puberty is delayed and/or growth continues into the early or mid-twenties, then an acceptable final adult height can be achieved. However, an individual’s maximum height may also be limited by genetic imprinting in very early development. This may be the case where full catch-up appears to have taken place but is followed by an advanced puberty and early cessation of growth [25–27].

One study showed that children with rickets on arrival did not achieve the recovery levels of non-stunted children, suggesting that deficiencies in early life can only be partially recovered. Such auxological dynamics can also be associated with early pubertal development which can shorten the period of growth and therefore contribute to a reduced adult height. Mechanisms underlying early pubertal development and optimal management of nutritional rehabilitation after chronic malnutrition need to be clarified [8]. One study, involving 107 girls, evaluated the model of growth and final height in relation to nutritional status at arrival and at menarche. The data suggest that most of the girls recovered some of their growth in height and about half of them in weight. The presence of faster recovery growth and a later arrival age were associated with an early menarche. Recovery growth was, however, incomplete, suggesting that linear growth and final height were influenced by pre-adoption nutritional conditions, as well as by the degree and timing of subsequent recovery growth and puberty times [27].

The development and growth of internationally adopted children in the countries of their adoptive families [28] should be carefully monitored. Unfortunately, many country-specific growth charts are out-of-date, were drawn up using small sample sizes and may be relevant to ethnic groups other than that of the adopted child. There was a step forward in 2006 when the World Health Organization released new international charts based on many thousands of children from Brazil, Ghana, India, Norway, Oman and the USA [29]. These Growth Standards have been adopted by many countries as a standard measure for assessing the growth of infants and young children [30, 31]. This is important because children from some ethnic groups could paradoxically present an excessive or reduced stature compared to their peers in their countries of arrival that is non-pathological and should not be treated as a medical condition.

Since pathological conditions may radically alter bodily growth, accurately estimating a child’s age is important in the care of adopted children, even in cases in which a child’s age appears undisputed [32]. The use of specific growth curves and reliable skeletal age detection techniques, together with a correct auxological evaluation, can bring to light an incorrect age and thus avoid social and psychological problems linked to an inappropriate collocation in school [32].

Finally, there has been discussion about the possible role of organic pollutants on pre-adoption growth failure. The little available data seems show that exposure to endocrine disruptors, air pollution, second-hand smoke, and the mother’s lifestyle during pregnancy affect children’s growth and development [33, 34]. Some studies have also suggested that endocrine-disrupting chemicals can affect the catch-up growth and early puberty of children who were born and exposed to such chemicals in developing countries and who subsequently moved to a developed country [33]. For example, a retrospective study involving 145 patients in Belgium who were treated for precocious puberty, suggested that in immigrant children (adopted and non-adopted), exposure to oestrogenic endocrine disrupters may have contributed to early puberty [33].

There is evidence that physiological adaptations to deprived environments in early childhood can increase the risk of obesity, early onset puberty in girls and metabolic syndrome and cardiovascular disease, particularly if there is a shift over time from resource-poor to resource-rich environments [35]. Clinically, there is concern that PI (post-institutionalized) children with rapid catch-up growth may be at a higher risk for obesity, early onset puberty in girls, and, in adulthood, metabolic syndrome [35].

Studies of children in low and middle income (LAMI) countries show that early postnatal catch-up growth is associated with obesity and a high risk of developing metabolic syndrome later in life. The rapid catch-up growth observed in internationally adopted children could be a risk factor for later obesity and early onset puberty, both of which are risk factors for cardiovascular diseases, type-2 diabetes, and musculoskeletal disorders [35]. However, one study which aimed to test the hypothesis that a shift from psychosocial and physical deprivation to highly resourced homes would increase the risk of obesity in boys and girls, suggested that stress and growth stunting early in life do not necessarily result in early onset puberty (for girls) and obesity [35]. It is possible that the early timing of removal from adversity or the context of the adoptees’ upbringing in highly resourced homes with highly educated parents may alter and perhaps buffer children from long-term impacts on BMI and early pubertal development. Of course, it is critical to follow the subsequent waves of data collection on this project to understand growth curves both of body fat and pubertal stage as the PI youths progress through adolescence [35].

### Puberty and pubertal disorders

The association between precocious puberty and international adoption was first described by Swedish authors in 1981 [36] in a case series of 7 girls adopted from India and Bangladesh and is well documented [37, 38]. Various studies in different countries have demonstrated the correlation [39, 40], although a recent report by Hayes et al. did not confirm the link in a population of 814 Chinese girls adopted in the USA and Canada [40].

There seem to be several causes for this association which are not fully understood. It is speculated that improved nutritional and socio-economic conditions lead to a period of catch up growth and the following early onset of puberty [37]. A similar phenomenon has been observed in the general population, where historical trends demonstrate that earlier puberty is a result of socio-economic development [41].

Some authors have postulated that early puberty follows the period of catch-up growth only after fetal or fetal-postnatal undernutrition, but not after postnatal undernutrition [41]. This is similar to what is described in many studies about IUGR and SGA children [42, 43].

Moreover, early puberty is also observed in nondeprived migrating children [42]. In defining early puberty, it is also important to establish the median age at menarche in a child’s country of origin and in cases where the age of a child cannot be accurately established it may be impossible to determine whether puberty is early [43].

The current treatment of idiopathic precocious puberty is with gonadotrophin-releasing hormone (GnRH) agonists [37]. Many studies have been published about the indications, goals and effects of this therapy but only a few concentrate on adopted children [37, 44, 45]. The main goals of therapy are auxological and psychological. For adopted children, it may often be impossible to establish a genetic target. Reference growth charts for the child’s native countries should be used. Predicting adult height in adopted children is difficult because it is difficult to know the long-term effects of early life adversity and malnutrition [44]. In general, responses to GnRH agonists in adopted children are similar to those of the general population with idiopathic precocious puberty [37]. Some reports evaluated the addition of GH to GnRH agonists with contrasting results [41, 44–46]. Mul et al. concluded that the association resulted in limited further increases in height gains [47].

### Thyroid disorders

Thyroid function is critical for the development of the central nervous system in the fetus and in children and iodine is an essential component of thyroid hormones [48]. 28.9% of the world’s population has an iodine deficiency [49] [Table 2]. In 2013, school-aged children in nearly 30 countries were deemed iodine-deficient, in 9 moderately deficient and in 21 mildly deficient [50]. Congenital hypothyroidism (CH) is the one of the most common preventable causes of mental retardation and the condition is included in programs of neonatal screening worldwide [51].

**Table 2:** The extent of congenital hypothyroidism screening worldwide

**Nord America**			
Full coverage of the population.			
**Latin America**			
Full coverage of the population with the exception of:			
Dominican Republic	Offered in the private sector		
El Salvador	Not available to all		
Haiti	–		
Honduras	–		
**Europe**			
Full coverage of the population with the exception of:			
Albania	Beginning implementation		
Azerbaijan	No data		
Kosovo	Beginning implementation		
Moldova	–		
**Middle East and North Africa**			
Available data about the coverage of the population:			
Algeria	Private efforts	Saudi Arabia	> 90%
Bahrain	> 90%	Somalia	–
Egypt	> 90%	Palestine	> 90%
Iran	85%	Sudan	–
Iraq	20%	Syrian	–
Israel	> 90%	Tunisia	
Jordan	> 90%	United Arab Emirates	100%
Kuwait	High coverage	Yemen	–
Lebanon	50%	Lybian	–
Oman	> 90%	Morocco	Starting
Qatar	100%		
**Asia Pacific**			
Full coverage of the population with the exception of:			
Cambodia	Pilot testing or not full population testing		
India	Pilot testing or not full population testing		
Indonesia	Pilot testing or not full population testing		
North Korea	Not available data		
Laos	Pilot testing or not full population testing		
Mongolia	Pilot testing or not full population testing		
Myanmar	Pilot testing or not full population testing		
Nepal	Pilot testing or not full population testing		
Pakistan	Pilot testing or not full population testing		
Sri Lanka	Pilot testing or not full population testing		
Vietnam	Pilot testing or not full population testing		
Modified by 51.			

Unfortunately, the spread of neonatal screening as well as campaigns for reducing iodine deficiency have obtained variable results and large areas of the planet are still not fully covered [Table 2]. It is therefore important to consider iodine deficiencies in adopted children.

Severe iodine deficiency may cause hypothyroidism that results in impaired somatic growth and motor development [51]. Overt cretinism is the most extreme form of mental retardation due to this deficiency [50].

In neonates of the Democratic Republic of the Congo, a 10% rate of biochemical hypothyroidism has been found [52]. This hypothyroidism persists into infancy and childhood if the deficiency is not corrected, and results in retarded physical and mental development [53]. In the most severely iodine deficient environments of Northern India, the incidence of neonatal hypothyroidism was 75 to 115 per thousand births [54]. By contrast in Delhi, where only mild iodine deficiency is present, the incidence drops to 6 per thousand [55].

Most neonates with CH have a normal appearance and no detectable physical signs at birth; hypothyroidism in this period is often overlooked and delayed diagnosis may to lead to mental retardation [51]. Pilot CH screening programs began in 1972 and new born screening is now routine in the developed world, but many countries still do not have effective screening programs [51]. Adoptees from countries were screening programs are not established should be screened for CH and if required undergo thyroid function examinations. The screening of iodine deficiency is also important and knowledge of iodine deficiency areas in the world is important for an early determination of this problem.

### Bone metabolism and density

Vitamin D deficiency is not only reported in cold or temperate countries [56], but also in countries with adequate sunshine [57]. Deficient calcium intake is also important [56, 57]. Adolescents are more prone to vitamin D deficiency because of the greater mineral demands of their growing skeletons [58]. Nutritional rickets in adolescents in temperate and tropical countries has been reported [59], even if the signs and symptoms may be subtle and nonspecific [60].

Vitamin D insufficiency is common in internationally adopted children, from all ethnic groups, although rickets has been cited only rarely in articles about the health problems of children adopted internationally during the 1980s and 1990s [60–62]. In children adopted from Romania, many of whom lived in orphanages for years before adoption, rickets has been mentioned only seldomly [63]. Early case reports of children with vitamin D deficiency rickets adopted from the former Soviet Union illustrate the physical findings that may be observed, some of which may be atypical [62]. It is reported that these children had limited sunlight exposure and no vitamin supplementation [62]. Many of the rachitic features typically resolve over 12–24 months with vitamin D and calcium supplementation. Vitamin D abnormalities are found in 46.7% of adopted children [64]; this deficit may be due to several factors, such as a low intake of calcium and vitamin D, environmental causes such as a lack of exposure to ultraviolet rays and ethnical racial characteristics such as the amount of melanin in the skin [65, 66]. Additionally, many children have inadequate caloric and/or vitamin D intake prior to adoption. A low body mass index and longer time spent in an institution (correlated to reduced time outdoors) are associated with vitamin D deficiency and insufficiency [67]. The significance of vitamin D insufficiency on bone development during the typical catch-up growth following international adoption needs to be determined [67].

Because children generally experience rapid catch-up growth in the first year with their adoptive families, they may need larger amounts of dietary or supplemental iron and other nutrients than other children of the same age. An evaluation of serum 25(OH) D levels after adoption can allow the prompt introduction of vitamin D supplementation/treatment if needed [68]. In internationally adopted children a specific therapy as well as prophylaxis for hypovitaminosis D should also be considered.Salerno and al have suggested that there is a widespread global vitamin D insufficiency and that higher doses than currently recommended are needed to reach country specific “desirable” levels. The early introduction of vitamin D supplementation/treatment may be particularly important in older children with dark skin who arrive in Italy during winter and spring [69].

### Hypothalamic-pituitary-adrenal (HPA) axis

Development of the hypothalamic-pituitary-adrenal axis is affected by genetics and pre- and postnatal environmental factors. A lot of studies report significant variations from normal functioning of the HPA caused by adversity early in life or even prior to conception [70]. These effects can lead to increased risks for mood and health disorders [71].

Studies about early life adversity (ELA) and HPA functioning provide discordant results [72]. Some studies identify hyper- and others hypo- or normal functioning of the axis, but the outcome measure is different varying from evaluation of basal to stress reactivity of the HPA axis. Subjects, ages, adversities and experimental paradigms are very heterogeneous [70] but most studies report a lower morning, flatter circadian cortisol rhythm [72] and down-regulation of the HPA axis also under stress conditions [73]. We should also consider children with prenatal alcohol exposure (PAE) because development of the HPA axis is shown to be influenced by PAE in animal models and human infants. If alcohol exposure is elevated (rank 4) children have cortisol levels that are significantly higher in the afternoon and at bedtime compared with control children or children with exposure to low or unknown levels of alcohol (alcohol exposure rank 3) [74]. These effects are long-term and could result in behavioural or emotional problems, vulnerability to mental health issues and diseases later in life [75]. Adopted children have a higher risk of ELA and have suffered from PAE more frequently than the general population. Significantly more elevated cortisol levels in the evening and flatter diurnal slopes were observed in children with both ELA and PAE than controls. It is likely that ELA and PAE negatively impact HPA development and functioning [75].

Some interesting studies have evaluated the potential for recovery of ELA damaged HPA, following improvements in care. Foster care or targeted interventions appear to be able to re-modulate HPA long-term [76]. The findings of DePasquale et al. offer initial evidence that post-adoption parental sensitivity may help promote internationally adopted children’s stress-regulatory development following severe early life adversity. Post-adoption parental sensitivity may be a vital resource through which internationally adopted children achieve positive developmental outcomes despite experiencing considerable early life adversity [77]. However, a recent study by Koss KJ et al. which examined changes in HPA in the two years after adoption, revealed persistently blunted HPA stress responses regardless of the child’s age at adoption (before or after 2 years) [78].

## Conclusion

In internationally adopted children disorders of linear growth, puberty development, thyroid function, and bone metabolism have been frequently reported. A careful auxological and endocrinological evaluation with follow-up is mandatory in these children. Paediatricians and other healthcare providers should be aware that auxological and endocrinological problems are common in newly arrived international adoptees and should tailor their approach to caring for these children accordingly.

## Data Availability

Not applicable.
